# Improving protein structure similarity searches using domain boundaries based on conserved sequence information

**DOI:** 10.1186/1472-6807-9-33

**Published:** 2009-05-19

**Authors:** Kenneth Evan Thompson, Yanli Wang, Tom Madej, Stephen H Bryant

**Affiliations:** 1 National Center for Biotechnology Information, National Library of Medicine, National Institutes of Health, 8600 Rockville Pike, Bldg. 38A, Bethesda, MD, USA; 2 Current address: Massachusetts Institute of Technology, Department of Biology, 400 Main St, Cambridge, MA, USA

## Abstract

**Background:**

The identification of protein domains plays an important role in protein structure comparison. Domain query size and composition are critical to structure similarity search algorithms such as the Vector Alignment Search Tool (VAST), the method employed for computing related protein structures in NCBI Entrez system. Currently, domains identified on the basis of structural compactness are used for VAST computations. In this study, we have investigated how alternative definitions of domains derived from conserved sequence alignments in the Conserved Domain Database (CDD) would affect the domain comparisons and structure similarity search performance of VAST.

**Results:**

Alternative domains, which have significantly different secondary structure composition from those based on structurally compact units, were identified based on the alignment footprints of curated protein sequence domain families. Our analysis indicates that domain boundaries disagree on roughly 8% of protein chains in the medium redundancy subset of the Molecular Modeling Database (MMDB). These conflicting sequence based domain boundaries perform slightly better than structure domains in structure similarity searches, and there are interesting cases when structure similarity search performance is markedly improved.

**Conclusion:**

Structure similarity searches using domain boundaries based on conserved sequence information can provide an additional method for investigators to identify interesting similarities between proteins with known structures. Because of the improvement in performance of structure similarity searches using sequence domain boundaries, we are in the process of implementing their inclusion into the VAST search and MMDB resources in the NCBI Entrez system.

## Background

As the amount of diverse biological data continues to grow, it is important for new methods of analysis to be devised and current methods to be improved. The ability to detect that two proteins have diverged from a common ancestor allows one to infer functional similarity between the two. A common method for identifying similarity between proteins is the use of sequence alignment tools such as FASTA [1] and BLAST [2], which provide an alignment of two sequences and a score indicating whether the alignment is significant or could be attributed to chance. The comparison of protein structures allows one to peer back farther into evolutionary time, based on the concept that a form or structure remains similar long after sequence similarity has become undetectable [3-6]. There are many methods [7-15] and databases [16-19] currently available for protein structure comparisons. While the performance of the methods and databases available are for the most part satisfactory, it is not unusual for such methods to miss certain biologically related protein structures that may be identified by human inspection. One may consider two directions when attempting to improve the ability to detect structural similarity. The first is to improve the similarity search method itself, either by using a novel approach for constructing an alignment or by optimizing an existing method. The second approach is to improve the definition of the objects to be compared by the methods. Although initial reflection on the two possibilities may indicate the first may be most fruitful, there is indeed a great deal that may be done with the data itself.

It has long been understood that there is an intermediate organization in proteins, typically called a domain, that is greater than secondary structure and less than the full-length chain of amino acids [20-22]. This fact considerably complicates the problems of sequence and structural alignment, because it is possible that two long proteins may contain a similar common domain, which is much smaller than either of the entire proteins. Ideally we want to recognize this situation, but it is difficult to detect true similarity of small subregions while at the same time excluding the small similarities that may occur due to chance. One part of the solution lies in testing for statistical significance of alignment scores or various similarity measures; but even so, it is possible for small but important similarities to be missed. Another part of the solution, which is possible in the case of structure comparison, is to identify the smaller subregions of potential similarity (the domains) and to directly compare them.

Thus, it becomes critical to identify the domains appropriately before performing structure similarity searches. Structurally compact domains are currently being used for computing related structures in MMDB. Recent studies investigating the performance of several structurally based domain parsers in comparison to expert curated structure domain boundaries have indicated the limitations of different methods and potential improvements [23,24]. Here we ask the questions, "How often do structurally identified domain boundaries disagree with those determined by sequence conservation" and "Does either domain type perform better in structure similarity searches when disagreement occurs?"

**Figure 1:**
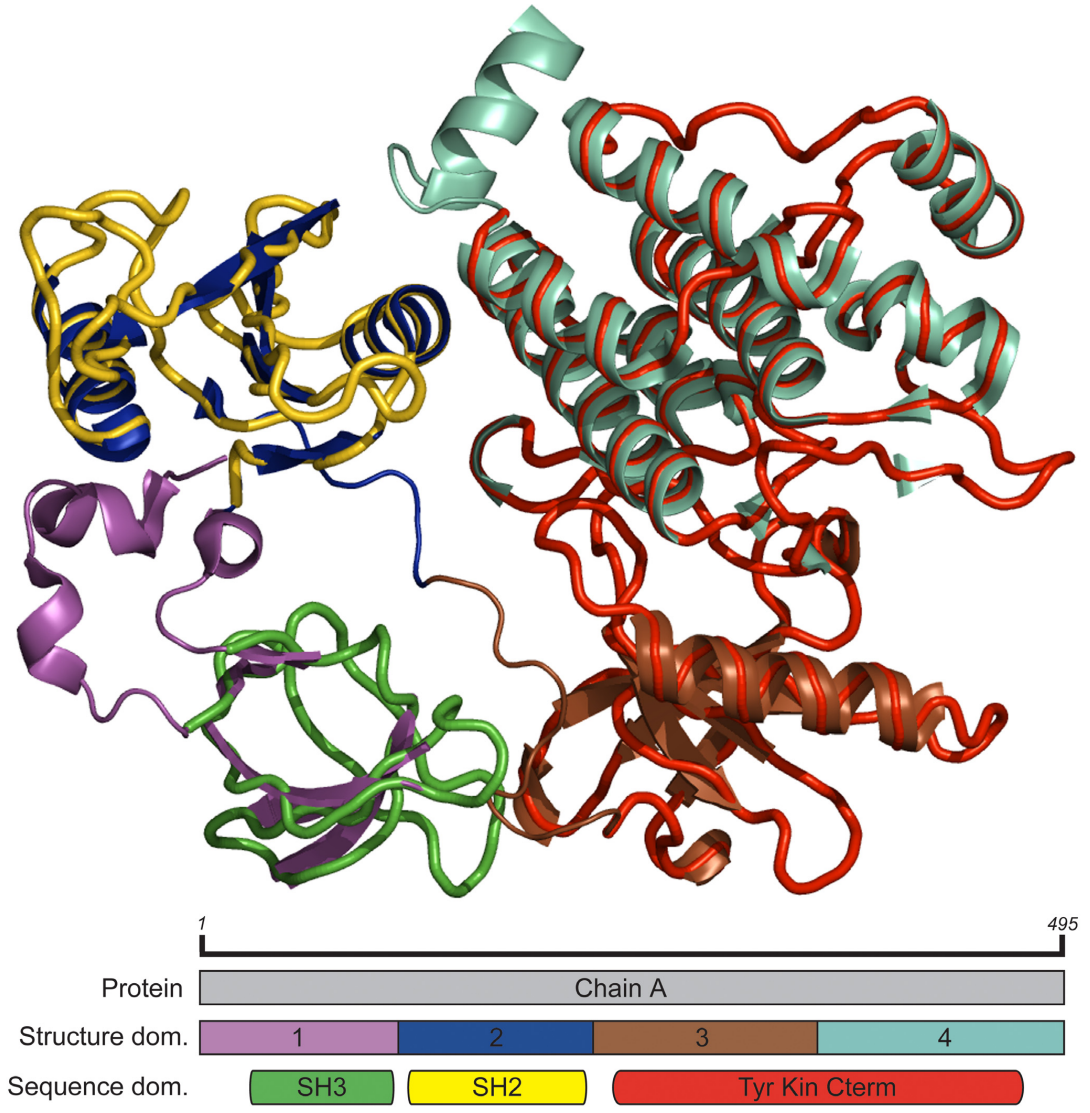
**Example of sequence/structure domain disagreement due to difference in concept**. The structure of ABL1 tyrosine kinase (PDB id: 2FO0, chain A) and the sequence domains SH3, SH2, and tyrosine kinase C-terminal region (CDD ids: cd00174, cd00173, cd00192). Structure based domains shown as cartoons in purple, blue, brown and light green. Sequence based C-terminal tyrosine kinase domain shown in red, SH3 shown in dark green, SH2 shown in yellow represented as thick backbone trace. The schematic of domain arrangement shows full protein chain, structure domains 1 through 4, and the three sequence domains.

In this study, taking advantage of the availability of large collections of manually curated domains based on conservation among sequences across a protein family, we investigated how the structure search performance of VAST would be affected when using sequence-based and structure-based protein domains. A sequence-based domain can generally be defined as an evolutionarily conserved region of a protein. Domains of this type are identified as similar blocks of residues occurring in several proteins. Currently there are many databases of these domains such as Pfam[25], SMART[26,27], and the Conserved Domain Database (CDD) [28,29], generally built up from multiple sequence alignments and hidden Markov model methods. A structure-based domain can be defined as a three dimensionally isolated region, and is considered by some to correspond to a compact folding unit. Structure-based domains are generally identified by manual inspection of a protein structure, as is the case with the Structural Classification of Proteins (SCOP) database[17], or by computationally delineating compact substructures as is done in the MMDB. Although in most cases the domains that are derived from sequence and structural ideas are consistent, there are times when the boundaries do not agree. An example of a common domain boundary disagreement can be seen in human ABL1 tyrosine kinase (PDB id: 2FO0, chain: A) (Figure 1) and is observed in many kinases. In this instance, the MMDB structure domain parser has divided the chain into four domains, as there are four geometrically distinct regions. However, two of the structure domains occur together with similar residue content across a diverse range of species in sufficient instances to be identified as a single domain, the tyrosine kinase catalytic domain (CDD id: cd00192), based on sequence analysis. In this case, we would want to investigate if combining both structure-based domains into a single domain would allow for detection of similarity to other kinases while avoiding detection of similarity to unrelated structures where smaller regions share a common arrangement of helices and strands.

In this work, we first systematically compared the domain boundaries of the sequence-based domains in the Conserved Domain Database to the structure-based domains in the medium redundancy subset of MMDB. We have identified a noticeable fraction of sequence based domains that differ significantly from those derived based on structural compactness. The new domains were then used as queries in identifying related structures using VAST and changes in structure similarity search results were analyzed. Using SCOP as a standard of truth, interesting cases were observed where the new domain boundaries perform better than the original domains in terms of homologous structure recognition. We have also found that the overall performance of sequence domains is comparable to that of whole chain and structure domain based queries.

## Results and Discussion

### Comparison of structure similarity search results

Having tested a series of thresholds for identification of differences between sequence and structure-based domains as described in the methods section, we focused on the structure similarity search results using sequence based domains for which at least 90% of the domain consensus sequence was aligned to a structure. A sequence based domain was determined to be different from existing structure based domains when its secondary structure composition was at least four secondary structure elements (SSEs) different from both the most similar structure domain and the entire protein chain. These results are derived from applying the methods to the medium redundancy subset of MMDB, in which the structure database is reduced by clustering similar structures based on sequence similarity and then selecting a single representative from each cluster based on structure quality [30]. We report our analysis results based on the non-redundant data set, though application of the methods and analysis to the larger non-identical subset of MMDB yielded similar performance of structure similarity searches. In addition, although we present the full analysis on older versions of the databases, recalculations using more recent version of the databases revealed similar ratios of domain differences found. The search for domain differences on the 6231 chains in the medium redundancy set identified 635 sequence domains on 495 protein chains. Of the differences found, one-third of the sequence domains fall within single structure based domain, whereas the remainder join regions of multiple structure domains.

Since this study is looking at the performance of different queries using the same search method and database, search performance can be assessed simply by comparing the ratio of homologs identified to all similarities identified for the two query sets, the original structure based domains and chains and the new sequence derived domains. The sequence-based domains do perform slightly better as queries than the original domains, having a search result set consisting of 21% homologous structures as opposed to 15% for the original domains (Table 1). Since one of the benefits of structure similarity searches is the ability to detect similarity at larger evolutionary distances and lower sequence similarity, we also used the same ratio method to assess performance with regards to the results falling in different sequence similarity groups. The result sets for each query type were divided into two bins, search hits with percent sequence identity greater than 20% against the query domain sequence and those with less than 20%. Again, sequence domains are performing slightly better based on the homolog ratios, with the hits having greater than 20% sequence identity consisting of 75% homologs for the sequence based domains as compared to 65% for the structure domains, and hits with sequence identity falling below the "twilight zone" of 20% consisting of 16% homologs as compared to 11% (Table 1). This second set of ratios shows that the sequence domain queries are not simply identifying a greater ratio of homologous sequence similar hits, but are indeed allowing the detection of distant homologs with minimal sequence similarity to the queries. Inspection of the raw numbers as opposed to the ratios shows that the sequence domain queries have a result set less than half the size of the result set for the structure domains. It is expected that the primary cause of this reduction is the nature of the method used. In order to verify that the sequence domain were identifying new hits previously being missed, we tested the performance of structure searches using both the original structure domains that were overlapped by the sequence domain, as well as using the entire chain as a query for searches, as all of these have structure search results provided in MMDB. This results in a minimum of two comparable original structure queries for each new sequence domain tested, and should account for the raw increase in total number of structure search hits. Nevertheless, the sequence based domains detect roughly 10% more unique structure neighbors when considered as an additional domain resource. ROC curves (data not shown) based on the percent identity between the queries and results revealed very similar performance of the structure and sequence based domains. The comparison of ROC curves based on percent identity for the structure domain based results and the additional hits found by sequence domain similarity shows a slight increase in performance. This indicates that the addition of sequence domains to the MMDB/VAST service would not degrade the overall performance of the system, while detecting additional homologs.

**Table 1:** VAST Search results for domains with boundary conflict from the medium redundancy subset of MMDB.

	Structure Domains Total	Sequence Domains Total	Structure Domains (> = 20% Id)	Sequence Domains (> = 20% Id)	Structure Domains (<20% Id)	Sequence Domains (<20% Id)
Homologs Found	9790 (15%)	5476 (21%)	2592 (65%)	1814 (75%)	7198 (11%)	3662 (16%)
Total Hits	65150	25560	3994	2416	61156	23144

A primary goal of this study was to determine if providing an additional automated resource to investigators allowing structure searches using alternate domain boundaries when endpoint conflict occurs could be beneficial. Our interest is not to seek domain definition replacement, but rather to see whether additional biologically relevant insight can be gained by using additional automatically generated domain sets. The analyses do reveal interesting new similarities that justify the inclusion of the sequence domain search results as an additional domain resource within MMDB. In the following two examples, we explore the scenarios of how structure domain and sequence domain can differ and the effects of the new boundaries on structure similarity search results.

### DNA topoisomerase I

**Figure 2:**
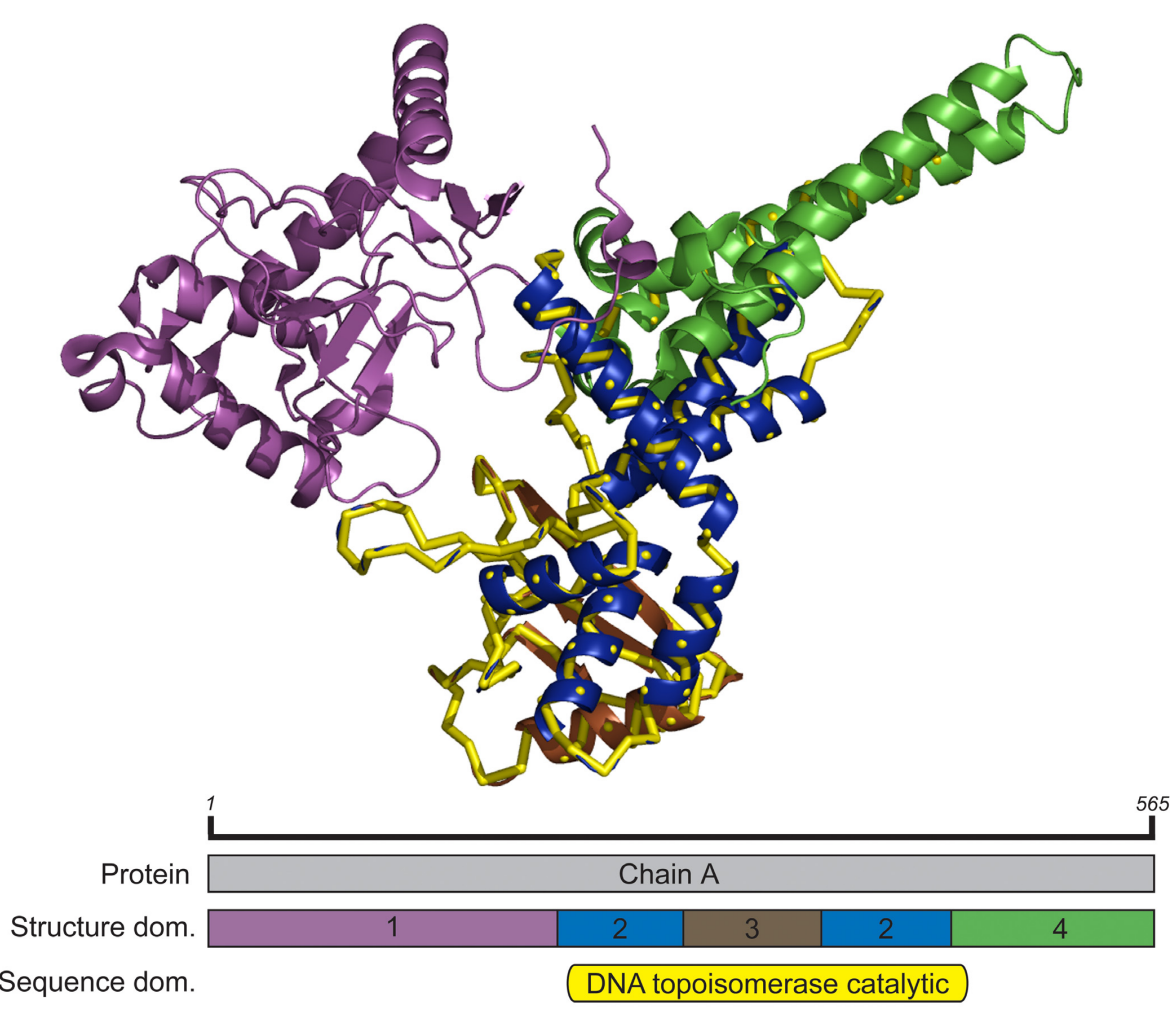
**Domain boundaries in human DNA topoisomerase I**. DNA topoisomerase from human (PDB id: 1RRJ, CDD id: pfam01028). Structure domains 1, 2, 3, and 4 are shown in magenta, blue, brown, and green in the structure and domain arrangement schematic, the DNA topoisomerase catalytic sequence based domain is shown in yellow.

The sequence based eukaryotic DNA topoisomerase-I catalytic core domain (CDD id: pfam01028) covers residues 231 to 466 which stretches across structure domains 2, 3, and 4 of human DNA topoisomerase-I (PDB id: 1RRJ, chain A) (Figure 2). The sequence domain consists of 8 helices and 5 strands, which includes all SSEs of the original structure domains 2 and 3, and a single helix of structure domain 4. This appears to be a case where the structure domain parser is excessively splitting the protein chain, and based on conserved sequence information, regions of several structure domains generally occur together. Inspection of other human topoisomerase-I structures reveals all have been structurally parsed with similar domain arrangements where the sequence conservation of the catalytic core domain covers a discontinuous structure domain and a small sequentially internal domain. By using the sequence domain boundaries, the only homolog detected by the original domains is found as well as two additional remote homologs (Table 2). We anticipate the same effect would be observed for other DNA topisomerase-Is which have similarly parsed structure domains. This example shows that the altered SSE composition associated with the sequence based domain allows for new 'hits' to be identified in the first VAST step of SSE vector alignment, thus helping to improve sensitivity in homolog recognition. In this example, not only does the sequence domain allow for the identification of new homologous structures, it also has the specificity benefit of eliminating the detection of similarity to all non-homologous structures previously found. The drastic increase in specificity led us to validate this performance gain by broadening our definition of homologous structures. We reanalyzed the search results of the structure domains using the SCOP fold group as our potential homolog set (Table 2). This verification did not result in the reclassification of any non-homologous similarities, confirming that in this case the alternative domain allows for the detection of additional homologous structures while eliminating detection of structures that do not share a common ancestor with the query.

**Table 2:** VAST Search results for Topoisomerase-I from human.

PDB Id and Chain	Structure Domain Hit	Sequence Domain Hit	Superfamily Member	Fold Group Member	SSEs Aligned	Residues Aligned	% Identity
1F34 B	+				4	32	13%

1HUM A	+				4	25	8%

1MGS A	+				4	24	13%

1ASS _	+				5	31	3%

2EOT _	+				4	22	0%

1EIG A	+				4	26	0%

1HFG A	+				4	23	22%

1AIH A	+	+	+	+	5 (9)	72 (126)	25% (9%)

1A41 _		+	+	+	(7)	(103)	(23%)

4CRX A		+	+	+	(9)	(139)	(12%)

Scores for sequence based domains shown in parentheses.

### Fibronectin

**Figure 3:**
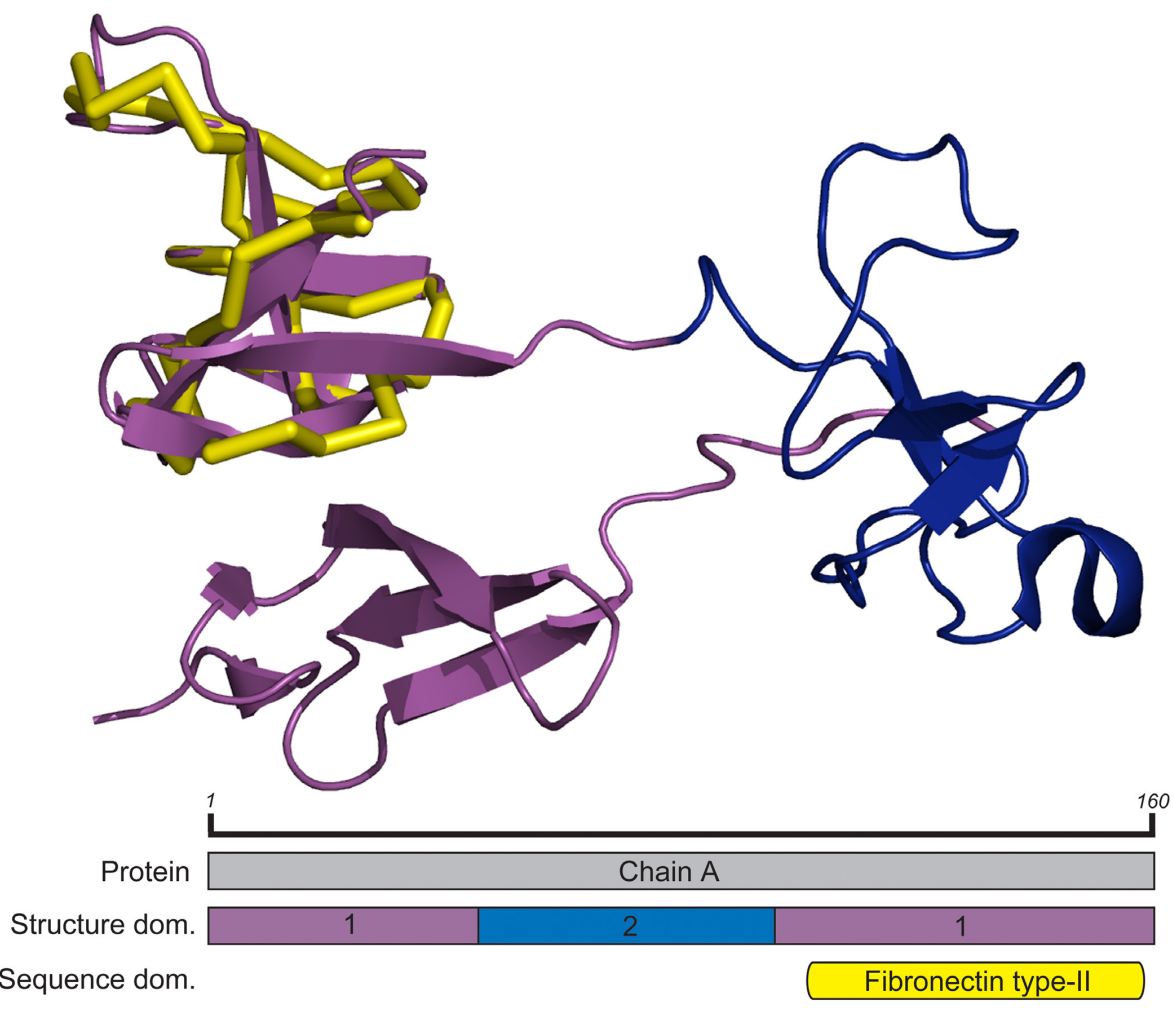
**Domain boundaries in human fibronectin**. Gelatin binding region from human fibronectin (PDB id: 1E88, chain A, CDD id: cd00062) Structure domains 1 and 2 are shown in magenta and blue in the structure and domain arrangement schematic, the fibronectin type-II sequence based domain is shown in yellow.

The sequence based Fibronectin type-II domain (CDD id: cd00062) eliminates the entire N-terminal region and reduces the C-terminal region of the discontinuous structure domain 1 of the gelatin binding region of human Fibronectin (PDB id: 1E88, chain A). This region covers residues 110 to 158 and consists of all but one of the 7 C-terminal strands (Figure 3). In this instance, it is easy to visualize how the parser judged the N- and C-terminal regions as packed together and distinct from structure domain two, although there is a visual distinction between the two discontinuous regions of domain 1. Inspection of the structure similarity search results shows searching with the individual fibronectin domain identified by sequence allows the identification of twice as many homologs at the cost of a single non-homolog based on SCOP superfamily and fold group classification, with some variability in the number of residues and SSEs aligned of common hits (Table 3). This increase in performance may be attributed to the fact that although in many cases individual fibronectin domains occur together, the way they pack together biologically or crystallographically may not be the same; thus searches with the individual regions are more advantageous in detecting similar domains in a variety of arrangements and conformations.

**Table 3:** VAST Search results for gelatin binding region of human fibronectin.

PDB Id and Chain	Structure Domain Hit	Sequence Domain Hit	Superfamily Member	Fold Group Member	SSEs Aligned	Residues Aligned	% Identity
2FN2 _	+	+	+	+	7 (5)	49 (39)	100% (100%)

1H8P A	+	+	+	+	5 (5)	34 (35)	41% (43%)

1PDC _		+	+	+	(5)	(27)	(48%)

1J7M A		+	+	+	(5)	(47)	(55%)

1MJD A		+			(4)	(17)	(12%)

Scores for sequence based domains shown in parentheses.

## Conclusion

Our investigation shows that although conflicting domain boundaries occur relatively infrequently, when disagreement occurs there is a slight gain in performance in the overall structure similarity search results by using sequence-based domain boundaries. While the improvement in performance is not consistently better for all differences identified, more structure neighbors are identified in general, and there are noticeable instances where there is a marked increase in the ability to distinguish homologs from non-homlogs in search results. As the number and quality of curated sequence conservation based protein domain families improves over time, the impact of sequence based domains on biologically related structure recognition could become more significant and it is clearly beneficial to add sequence based domains in automatic fashion when computing related structures in MMDB. We are in the process of implementing the inclusion of sequence domains into the protein structure resources in the Entrez system at NCBI. MMDB protein structure pages will soon allow for inspection of similar structures detected using sequence domains and the VAST search service will allow such sequence based domains to be identified automatically in user submitted structures, permitting these subregions to be used as queries for structure similarity searches. The addition of sequence domain boundaries to these services will allow investigators to potentially identify interesting new relationships between protein structures that were previously undetected, and similar screening methods could easily be applied to other search systems.

## Methods

### Identifying sequence domains disagreeing with structure domains

The April 2005 Conserved Domain Database (CDD) and medium redundancy Molecular Modeling Database (MMDB) were used for the sequence to structure domain comparisons, the current versions of which are available at  and . Of the Conserved Domain Database, entries derived from the Clusters of Orthologous Groups[31,32] database were excluded, as they tend to be full gene products containing multiple sequence and structure domains. Each domain profile from the CDD was compared to the sequence of protein structures in our subset of MMDB. These comparisons involved using the sequence of MMDB entries as queries in RPS-BLAST against our subset of the CDD, using a 'hit' expectation value threshold of 0.01 and a requirement that at least 90% of a Conserved Domain (CD) sequence be aligned to a query to be considered for comparison. We also tested the effect of reducing the percentage of CD sequence alignment required for boundary difference comparisons. Since these tests resulted in few additional domain identifications at the cost of reduced sequence alignment length, we focused on our most stringent 90% alignment coverage requirement. Because the CDD is collected from several database sources, some domains in the database are very similar, thus sequence domains are curated into domain families. When collecting the set of sequence domains, if multiple sequence domains from the same family aligned to a protein structure, a family representative was chosen based on the following criteria: 1) The domain family member with the greatest percentage alignment was chosen, and 2) if more than one domain family member had the same percentage alignment, the member with shorter overall length was chosen.

**Figure 4:**
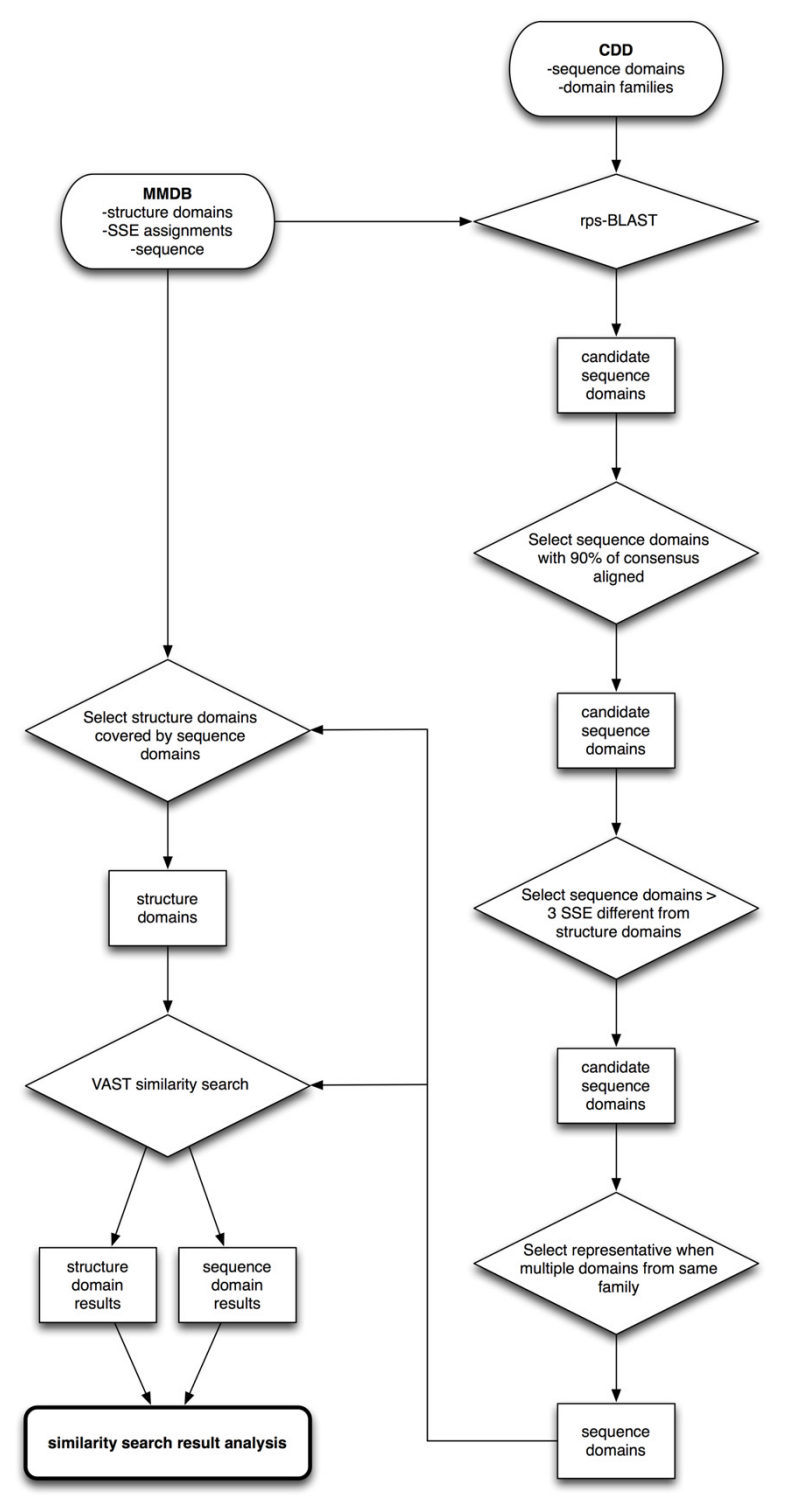
**Order of operations for identifying domain differences**. Databases are shown as squashed rectangles, results as rectangles, operations as diamonds, and endpoint as a rounded rectangle.

The SSE compositions of the sequence domains were then compared to the composition of the entire chain on which the sequence domain was identified, as well as the domains of the chain identified based on compactness. A SSE composition metric was used, rather than an amino acid sequence difference requirement, because the VAST algorithm applied later in the study uses SSE alignment to detect protein similarity. By requiring different SSE composition, we avoided the potential identification of domain differences resulting from the inclusion or removal of unstructured protein regions which would have no effect on structural similarity searches employed later in the study. The footprint of a sequence domain was considered different from the structure domains based on the following criteria: 1) The sequence domain must contain at least 4 secondary structure elements (SSEs), 2) the sequence domain must be at least 4 SSEs shorter than the whole chain, and 3) the sequence domain must be at least 4 SSEs different from the closest structure based domain. SSEs and structure domains for a given structure were those identified by the MMDB structure domain parser. Item 1) simply means that we are not considering very small domains with 3 or fewer SSEs, which corresponds fairly well to having 50 or fewer residues. Items 2) and 3) define when we consider a sequence domain to be "different" from a structure-based domain. Additional SSE difference requirements were tested, and as expected, reducing the number of SSEs required resulted in many domain differences identified, while more SSEs required for being classified as different quickly reduced the number of differences found. This testing led us to select the 4 SSE difference requirement as a "middle ground", allowing us to identify a large number of domain differences without selecting all sequence based domains in the structure database. The choice of 4 SSEs is natural, because this is about the size of a small domain, and it should allow us to clearly see the effect of using different domain boundaries in the structure similarity searches. The closest structure domain to a sequence domains was determined as follows: a) the structure domain completely covered by the sequence domain, b) the longest structure domain completely covered by the sequence domain, or c) the structure domain with the longest length covered by the sequence domain. To test the possibility that domain differences were due to unaligned ends of the sequence domains not being included in the regions to be used as queries, we repeated the method using the July 2005 databases in which the sequence domain boundaries were extended to the ends of the complete sequence domains and tested using the same SSE difference requirements described above. Differences in the size and composition of the footprint and extended footprint sets were then compared, revealing minimal differences in the domains identified as different. A flowchart of the order of operations can be seen in Figure 4.

### Structure similarity search assessment

The domain entries from MMDB and sequence domains identified as different were used as queries for structure similarity searches against the medium redundancy set of MMDB using Vector Alignment Search Tool (VAST), available on the web at . VAST is essentially a two-phase process, the first being the alignment of vectors of secondary structure and preliminary scoring. Those initial alignments whose scores exceed an empirically derived threshold are then refined in the second phase of structural alignment using the Ca coordinates. Only those refined alignments with a statistical significance of P < 10^-5 ^are reported as structurally similar. Although available to the public on the web, our study used an in-house version of the VAST executable to allow the submission of multiple queries and more efficient use of computational resources. To evaluate the change in structure similarity search results when using the new domains based on sequence, we considered structurally similar domains classified within the same superfamily division as the query domain of SCOP 1.69, available at , to be homologs. Since the study explicitly looked for differences in domain boundaries, it was not possible to directly map both structure and sequence domains to corresponding entries in the SCOP database. For example, if a structure domain from MMDB has very similar domain boundaries as a SCOP domain, then a sequence domain found to be different from the MMDB domain would also be different from a SCOP domain definition. Thus, in order to measure the ability to identify similar domains, a homolog set for a query domain was identified as the SCOP superfamily members for all SCOP domains identified on the query chain. Although this 'collapsing' of superfamilies on a chain could introduce the possibility of some false homolog mapping or unrealistically large homolog sets, it allowed for sensitivity and specificity analysis of individual domains in the test set as well as overall assessment of the domain based structure similarity search result sets. In addition, to avoid missing data issues due to the smaller size of the SCOP database, all domains used as VAST queries and resulting similar structures were reduced to only those structures included in the 1.69 release of SCOP. Individual search results were also evaluated using SCOP fold classification members to test the possibly that previously identified non-homologs were potentially distant homologous structures that were not included in the superfamily classification. The structure similarity search results for each domain query and domain type sets were then compared based on the homologous and non-homologous structures found, as well as search result overlap, e.g. hits common to both sequence and structure domain similarity search results, regardless of the significance scores of the alignment other than the statistical significance of P < 10^-5 ^required for being reported as similar by the VAST algorithm. Individual search results of the new domains were then compared to results of the original structure domains and visualized using PyMOL [33] and Cn3D [34].

## Authors' contributions

KET designed the experiments, performed the analysis, and wrote the manuscript. YW helped design the experiments, provided feedback throughout the study, and edited the manuscript. TM provided feedback throughout the study, assisted with VAST search setup, and edited the manuscript. SB conceived of the study and directed the work. All authors read and approved of the final manuscript.

## References

[B1] Pearson WR, Lipman DJ (1988). Improved tools for biological sequence comparison. Proc Natl Acad Sci USA.

[B2] Altschul SF, Gish W, Miller W, Myers EW, Lipman DJ (1990). Basic local alignment search tool. J Mol Biol.

[B3] Chothia C, Lesk A (1986). The relation between the divergence of sequence and structure in proteins. EMBO J.

[B4] Doolittle R (1981). Similar amino acid sequences: chance or common ancestry?. Science.

[B5] Sierk M, Pearson W (2004). Sensitivity and selectivity in protein structure comparison. Protein Sci.

[B6] Wood T, Pearson W (1999). Evolution of protein sequences and structures. J Mol Biol.

[B7] Gibrat J, Madej T, Bryant S (1996). Surprising similarities in structure comparison. Curr Opin Struct Biol.

[B8] Holm L, Sander C (1993). Protein structure comparison by alignment of distance matrices. J Mol Biol.

[B9] Jung J, Lee B (2000). Protein structure alignment using environmental profiles. Protein Eng.

[B10] Levitt M, Gerstein M (1998). A unified statistical framework for sequence comparison and structure comparison. Proc Natl Acad Sci USA.

[B11] Mooney S, Liang M, DeConde R, Altman R (2005). Structural characterization of proteins using residue environments. Proteins.

[B12] Shindyalov I, Bourne P (1998). Protein structure alignment by incremental combinatorial extension (CE) of the optimal path. Protein Eng.

[B13] Szustakowski J, Weng Z (2000). Protein structure alignment using a genetic algorithm. Proteins.

[B14] Taylor W (1999). Protein structure comparison using iterated double dynamic programming. Protein Sci.

[B15] Zhi D, Krishna S, Cao H, Pevzner P, Godzik A (2006). Representing and comparing protein structures as paths in three-dimensional space. BMC Bioinformatics.

[B16] Chen J, Anderson J, DeWeese-Scott C, Fedorova N, Geer L, He S, Hurwitz D, Jackson J, Jacobs A, Lanczycki C (2003). MMDB: Entrez's 3D-structure database. Nucleic Acids Res.

[B17] Murzin A, Brenner S, Hubbard T, Chothia C (1995). SCOP: a structural classification of proteins database for the investigation of sequences and structures. J Mol Biol.

[B18] Orengo C, Michie A, Jones S, Jones D, Swindells M, Thornton J (1997). CATH – a hierarchic classification of protein domain structures. Structure.

[B19] Pearl F, Todd A, Sillitoe I, Dibley M, Redfern O, Lewis T, Bennett C, Marsden R, Grant A, Lee D (2005). The CATH Domain Structure Database and related resources Gene3D and DHS provide comprehensive domain family information for genome analysis. Nucleic Acids Res.

[B20] Philips David C (1966). The three-dimensional structure of an enzyme molecule. Sci Am.

[B21] Edelman G, Cunningham B, Gall W, Gottlieb P, Rutishauser U, Waxdal M (1969). The covalent structure of an entire gammaG immunoglobulin molecule. Proc Natl Acad Sci USA.

[B22] Edelman G (1970). The covalent structure of a human gamma G-immunoglobulin. XI. Functional implications. Biochemistry.

[B23] Holland T, Veretnik S, Shindyalov I, Bourne P (2006). Partitioning protein structures into domains: why is it so difficult?. J Mol Biol.

[B24] Veretnik S, Bourne P, Alexandrov N, Shindyalov I (2004). Toward consistent assignment of structural domains in proteins. J Mol Biol.

[B25] Finn R, Mistry J, Schuster-Böckler B, Griffiths-Jones S, Hollich V, Lassmann T, Moxon S, Marshall M, Khanna A, Durbin R (2006). Pfam: clans, web tools and services. Nucleic Acids Res.

[B26] Schultz J, Milpetz F, Bork P, Ponting C (1998). SMART, a simple modular architecture research tool: identification of signaling domains. Proc Natl Acad Sci USA.

[B27] Letunic I, Copley R, Pils B, Pinkert S, Schultz J, Bork P (2005). SMART 5: domains in the context of genomes and networks. Nucleic Acids Res.

[B28] Marchler-Bauer A, Panchenko A, Shoemaker B, Thiessen P, Geer L, Bryant S (2001). CDD: a database of conserved domain alignments with links to domain three-dimensional structure. Nucleic Acids Res.

[B29] Marchler-Bauer A, Anderson J, Cherukuri P, DeWeese-Scott C, Geer L, Gwadz M, He S, Hurwitz D, Jackson J, Ke Z (2005). CDD: a Conserved Domain Database for protein classification. Nucleic Acids Res.

[B30] (2007). VAST Help. http://www.ncbi.nlm.nih.gov/Structure/VAST/vasthelp.html.

[B31] Tatusov R, Koonin E, Lipman D (1997). A genomic perspective on protein families. Science.

[B32] Tatusov R, Fedorova N, Jackson J, Jacobs A, Kiryutin B, Koonin E, Krylov D, Mazumder R, Mekhedov S, Nikolskaya A (2003). The COG database: an updated version includes eukaryotes. BMC Bioinformatics.

[B33] DeLano M (2002). The PyMol Molecular Graphics System.

[B34] Wang Y, Geer L, Chappey C, Kans J, Bryant S (2000). Cn3D: sequence and structure views for Entrez. Trends Biochem Sci.

